# The Hunt Opinion Model—An Agent Based Approach to Recurring Fashion Cycles

**DOI:** 10.1371/journal.pone.0166323

**Published:** 2016-11-11

**Authors:** Rafał Apriasz, Tyll Krueger, Grzegorz Marcjasz, Katarzyna Sznajd-Weron

**Affiliations:** 1 Department of Theoretical Physics, Faculty of Fundamental Problems of Technology, Wrocław University of Science and Technology, Wrocław, Poland; 2 Department of Control Systems and Mechatronics, Wrocław University of Science and Technology, Wrocław, Poland; 3 Faculty of Pure and Applied Mathematics, Wrocław University of Science and Technology, Wrocław, Poland; University of Manchester, UNITED KINGDOM

## Abstract

We study a simple agent-based model of the recurring fashion cycles in the society that consists of two interacting communities: “snobs” and “followers” (or “opinion hunters”, hence the name of the model). Followers conform to all other individuals, whereas snobs conform only to their own group and anticonform to the other. The model allows to examine the role of the social structure, i.e. the influence of the number of inter-links between the two communities, as well as the role of the stability of links. The latter is accomplished by considering two versions of the same model—*quenched* (parameterized by fraction *L* of fixed inter-links) and *annealed* (parameterized by probability *p* that a given inter-link exists). Using Monte Carlo simulations and analytical treatment (the latter only for the annealed model), we show that there is a critical fraction of inter-links, above which recurring cycles occur. For *p* ≤ 0.5 we derive a relation between parameters *L* and *p* that allows to compare both models and show that the critical value of inter-connections, p*, is the same for both versions of the model (annealed and quenched) but the period of a fashion cycle is shorter for the quenched model. Near the critical point, the cycles are irregular and a change of fashion is difficult to predict. For the annealed model we also provide a deeper theoretical analysis. We conjecture on topological grounds that the so-called saddle node heteroclinic bifurcation appears at p*. For *p* ≥ 0.5 we show analytically the existence of the second critical value of *p*, for which the system undergoes Hopf’s bifurcation.

## Introduction

Theories of social cycles are among the earliest theories in sociology, dating back to the seminal works of Vilfredo Pareto [1]. In this paper we focus on recurring fashion cycles, which are empirically documented in many studies [2–5] and can be described within several classical theories [6–8]. Among them, probably the most known, universal and influential is the one proposed by Simmel [8, 9]. As noted by Simmel “*For fashion to exist, society must be stratified, some members must be perceived as inferior or superior—or simply as worthy or unworthy of being imitated*”. Certainly not all fashion cycles can be described within Simmel’s theory [9–11]. A good example is the mid-20th century vogue for blue jeans—a phenomenon that, at least at its outset, was intended as a political statement and celebration of working people rather than, say, intra-group dynamics involving self-conscious contrariness. Moreover, two-population models are not necessary to describe recurring social cycles. For example, an interesting preference model, in which individuals are described by traits and preferences, has been introduced in [12] as an alternative to status models [6, 9] or the neutral model [13]. Nevertheless, Simmel’s theory can be applied to understand many fashion cycles [12, 14, 15] and is particularly interesting from the modeling point of view, due to its simplicity.

From a psychological point of view, Simmel’s theory is based on two types of social response—conformity (a result of the tendency to imitate others) and anticonformity (a result of the tendency to distinguish ourselves from others). It is well known that both types of social response are just two faces of the same coin. They are opposites at the operational level, but at the conceptual level the responses are similar in that both indicate behavior that has been influenced by the source [16]. This immediately implies the second similarity between both types of response—they are relative, which means that the same individual may conform to one group and anticonform to another.

While conformity underlies many models of opinion dynamics, including various Ising-type models (i.e., agent-based models with agents described by a single trait that takes only binary values, for reviews see [17–19]), anticonformity is a much less considered type of social response. In the context of binary opinion dynamics models, anticonformity was probably introduced for the first time in 2004 by Galam using the notion of ‘contrarians’ [20]. Later on, this idea has been applied to other Ising-type models (see a short review in [21]). However, to the best of our knowledge, the relativity of conformity vs. anticonformity has been considered for the first time only very recently, in a simple model of two interacting cliques [22]. The model has been built on a modified version of the *q*-voter model [23, 24], which occurred to be very interesting both from the theoretical [25–29] and the applicative [30–33] points of view. Using Monte Carlo simulations it has been shown that the interplay between intra-clique conformity and inter-clique anticonformity leads to a bi-polarized state of the entire system if only fraction *L* of cross links between cliques is larger than the critical value, L* = L*(*q*). For smaller values of *L*, consensus in the entire system is possible.

From the physical point of view, conformity corresponds to ferromagnetic interactions and anticonformity to antiferromagnetic, what has been already discussed in [21]. Therefore, the model introduced in [22] reminds of the Ising model with competing ferromagnetic interactions between nearest neighbors and antiferromagnetic between next nearest neighbors [34–36]. From this point of view, the existence of the critical value of the interplay between conformity and anticonformity, below which consensus exists, is not very surprising. On the other hand, the two-community structure with competing ferro- and antiferromagnetic interactions has not been investigated before and seems to be interesting for social applications. It is also worth to recall that the two-community structure has been already investigated in the context of the Ising model in [37, 38] and the linear voter model [39], but in all these papers only ferromagnetic interactions were investigated. Here we propose a modification of a model introduced in [22] that could not be easily motivated by any physical problem but is very interesting from the social point of view—we break the symmetry between cliques.

Our modification goes in line with fashion theories that assume an inequality between groups—members of the first group conform to all individuals (we will call them “followers” or “opinion hunters”, hence the name of the model), whereas members of the second group conform only inside their own group and anticonform to members of the other group (we will call them “snobs”). As we have mentioned, our modification seems to be bizarre from the physical point of view, because it would mean that interactions between particles are non-symmetric. Therefore, to the best of our knowledge, it has never been studied before within Ising-type models. As we will show, such a modification leads to a new type of behavior, that has not been observed in [22] and is very often found in fashion [2–5]—above a critical fraction of inter-links recurring cycles occur.

Although we are aware that our model is very simple and we do not claim that it describes recurring fashion cycles in all their richness, we hope that it makes a contribution to the understanding of social cycles for which inequalities between groups are important. Moreover, in spite of its simplicity it takes into account several empirically observed facts:

- Social networks display community structure—groups of nodes within which connections are dense but between which they are sparse and the number of inter-community links (edges) can be measured [40–42]. Here we use the double click approximation [39], which allows for analytical treatment and has been used also in the original model [22].
- People often select cultural tastes (opinions, attitudes, behaviors, etc.) that distinguish them from members of other groups. Moreover, they abandon cultural tastes when members of other social groups adopt them [11]. Therefore we use here the idea introduced in [22], which links the type of social response with the group identity.
- Empirical studies have shown that across different informal locations, freely-forming groups range in size from 2 to 7 with the average being 2.41 [43, 44]. This justifies the assumption that underlies the *q*-voter model that simultaneously each individual can interact only with a small group of *q* neighbors, instead of all his/her neighbors.
- The social pressure increases with the size of the group but a small unanimous group may be more efficient in convincing others than a much larger group with a non-unanimous majority [45]. This justifies the second assumption of the *q*-voter model that a unanimous opinion of the *q* neighbors is needed to influence others.

According to [6], our model belongs to the class of *true endogenous cycle models*, which means that all phenomena observed on the macroscopic scale will be driven by direct interactions between agents on the microscopic scale. In the Simmel theory it is assumed that members of the elite group abandon a certain fashion because too many others, lower status individuals, adopted this fashion. However, in our model we do not need to assume this—none of the individuals has knowledge of any global, aggregated variable, such as the average preference or opinion. The macroscopic “elite’s escape” will emerge spontaneously from direct interactions between the spinsons. Stressing the endogenous nature of the model, we do not claim that exogenous factors are not important for fashion. Public media or social norms are undoubtedly very important. For example, recently it has been shown empirically based on a large-scale dataset of individual purchases of women’s shoes (16,236 transactions) across five years that women conform to new local norms (i.e. the average heel size) when moving to relatively higher status locations, but mostly ignore new local norms when moving to relatively lower status locations [10]. Our claim is that we do not need to introduce any exogenous factors to obtain recurring cycles, a fact acknowledged in the population dynamics literature [46, 47].

We will examine here the role of the number of inter-links between two groups of agents, as well as their character. Therefore, we will consider two versions of the model—*quenched* (parameterized by fraction *L* of fixed inter-links) and *annealed* (parameterized by probability *p* that a given inter-link exists; on average the number of inter-links is constant but links can change randomly over time). We would like to stress here that with respect to fashion there is no need to interpret links between individuals in a traditional way, i.e. in terms of friendship, collaboration or even acquaintance. In fact, we even do not need to know the person to be influence by him or her. Within such a general interpretation, links can denote the channels of potential influence, which justifies consideration of very large cliques and the annealed approach. Moreover, the annealed approach allows for analytical treatment, which greatly facilitates the verification of an agent-based model [48].

The main message of this paper is the following—interconnections between two unequal groups (snobs and followers) are not sufficient to produce recurring social cycles. Periodic behavior appears only above a certain critical value of interconnections, which is in a sense universal, i.e. has the same value for the fixed (quenched) and evolving (annealed) social networks. As usual, near the critical state, above which recurring cycles appear, changes in fashion are strongly irregular and therefore hard to predict.

## Methods

We consider the following opinion formation model. There are 2*N* nodes divided into 2 cliques of size *N*. Each node is associated with one autonomous agent characterized at each time instant *t* by a dynamical binary variable *S*_*i*_(*t*) = ±1. This variable, depending on the context, can be interpreted as a dichotomous opinion (agree/disagree) or a choice between two alternatives, e.g. old product vs. innovation, long vs. short skirt, Samsung vs. Apple, red or white wine [49]. Since agents are described by a single binary variable we will also refer to them as *spinsons*, a term coined by Nyczka and Sznajd-Weron [21] that reflects the dichotomous nature of an agent (spinson = spin + person).

As is common in the literature, for the macroscopic quantity that describes the state of the system at time *t* we choose the public opinion (alternatively interpreted as the ratio of adopted [30] or the market penetration rate [31, 49]):

$$\begin{array}{c} m\left(t\right)=\frac{1}{2N}\sum_{i=1}^{2N}S_{i}\left(t\right). \end{array}$$ (1)

**Fig 1:**
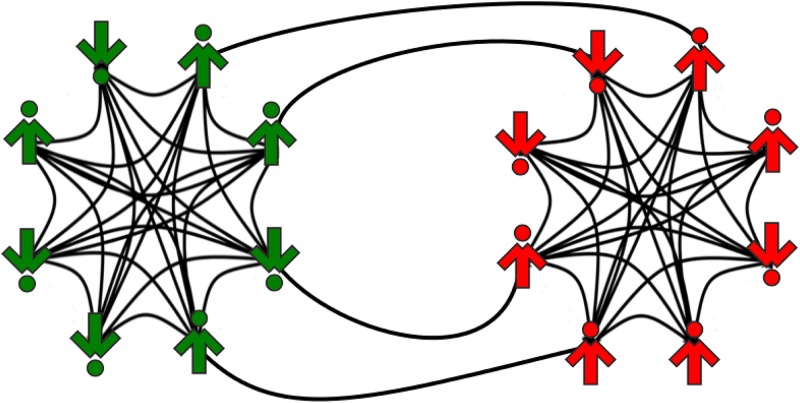
An example of a system that consists of two interacting cliques (complete graphs). The left clique (*σ* = 1) consists of “followers”, i.e. spinsons that behave like conformists with respect to both cliques, whereas the right clique (*σ* = −1) consists of “snobs”, i.e. individuals that behave like conformists with respect to their own group and anticonform to the other. In this example the cliques are of size *N* = 8, which means that the number of links inside each clique is equal to *N*(*N* − 1) = 56 and the number of all possible cross links is *N*^2^ = 64. Moreover, the number of cross links is 5, which means that the fraction of cross links *L* = 5/64 = 0.078.

Two cliques can interact with each other through cross links between them. The number of cross links is equal to *LN*^2^, where the fraction of cross links *L* ∈ [0, 1] is a parameter of the model, see Fig 1. In order to clarify the description of the model let us introduce a second variable: *σ*_*i*_ = ±1. It defines to which of the two cliques the *i*-th spinson belongs. In contrast to the opinion, *S*_*i*_(*t*), this variable is static, i.e. it does not change in time (*σ*_*i*_ = 1 if the *i*-th spinson belongs to the first group and *σ*_*i*_ = −1 if it belongs to the second clique). Within such a formulation we can separately calculate the opinions in each clique:

$$\begin{array}{c} m_{1}\left(t\right)=\frac{1}{N}\sum_{i=1}^{2N}\delta_{1,\sigma_{i}}S_{i}\left(t\right), \end{array}$$ (2)

$$\begin{array}{c} m_{2}\left(t\right)=\frac{1}{N}\sum_{i=1}^{2N}\delta_{-1,\sigma_{i}}S_{i}\left(t\right), \end{array}$$ (3)

where *δ*_*j*,*k*_ is the Kronecker delta, i.e. *δ*_*j*,*k*_ = 1 for *k* = *j* and 0 elsewhere. The summation in the above formulas is taken over the entire system, i.e. over 2*N* spinsons. However, the maximum value of each sum is *N*. This is guaranteed by the Kronecker delta: for *m*_1_(*t*) only spinsons from the first clique will contribute to the sum and analogously for *m*_2_(*t*) only spinsons from the second clique will contribute to the sum. Therefore we use factor 1/*N* in front of each sum.

In [22] it has been assumed that every spinson behaves like a conformist inside its own clique and anticonforms with respect to agents in the other group. Both types of social response have been described within the generalized *q*-voter model [21], which means that a spinson responds to the social influence only if the *q*-panel, which consists of *q* randomly chosen neighbors (spinsons that are linked to a given spinson), is unanimous. In case of conformity a spinson takes the same opinion as the *q*-panel, whereas in the case anticonformity it takes the opposite opinion.

This idea can be easily described using *σ*: if the *i*-th spinson is the one to be influenced (the so-called *target* in theories of social response [16, 32]) and the *j*-th spinson is the source of influence, then the *i*-th spinson conforms to opinion *σ*_*i*_ *σ*_*j*_ *S*_*j*_(*t*), which equals *S*_*j*_(*t*) if spinsons *i* and *j* belong to the same group (*σ*_*i*_ *σ*_*j*_ = 1) and −*S*_*j*_(*t*), i.e. anticonformity, if they belong to different groups (*σ*_*i*_ *σ*_*j*_ = −1). The *i*-th spinson having opinion *S*_*i*_(*t*) at time *t* is influenced by the group of *q* neighbors *S*_1_(*t*), …, *S*_*q*_(*t*) if the local average opinion:

$$\begin{array}{c} M_{i}\left(t\right)=\frac{1}{q}\sum_{j=1}^{q}\sigma_{i}\sigma_{j}S_{j}\left(t\right)=\pm 1. \end{array}$$ (4)

If *M*_*i*_(*t*) = 1 then *S*_*i*_(*t* + 1) = 1 and if *M*_*i*_(*t*) = −1 then *S*_*i*_(*t* + 1) = −1.

Within the model proposed in [22] both cliques are equivalent. In this paper we break the symmetry between the cliques and assume that a spinson which belongs to group 1 (*σ* = 1) conforms to all other spinsons. This means that—using the notation from the previous paragraph—the *i*-th spinson always conforms to opinion *S*_*j*_(*t*), not to *σ*_*i*_ *σ*_*j*_ *S*_*j*_(*t*).

### A model with quenched cross links

**Fig 2:**
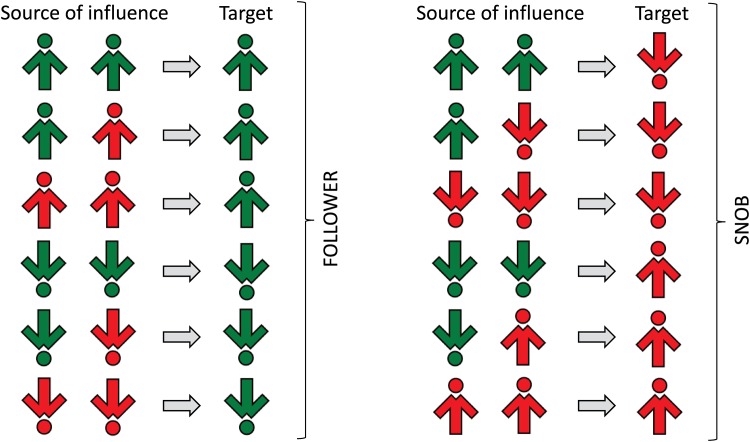
All possible transitions in the hunt opinion model for *q* = 2. Social responses of an influenced “follower” (i.e. a spinson from the first clique; green color) are shown in the left panel, whereas the right panel presents the behavior of an influenced “snob” (i.e. a spinson from the second clique; red color). Only transitions that change the state of the system are shown.

For each simulation we build a new network with the same statistical properties, i.e. two cliques of size *N* connected by *LN*^2^ cross links. The network does not change in time, i.e. it is a “quenched network”. A single Monte Carlo update for the model with quenched links is described by the following algorithm (see Fig 2):

1. Randomly choose one spinson, say the *i*-th spinson, from the 2*N* spinsons in the system; goto 2.
2. Build the influence group by randomly choosing *q* spinsons from all those that are linked to the *i*-th spinson (*q*-panel): *S*_1_, …, *S*_*q*_; goto 3.
3. If the *i*-th spinson belongs to clique 1 then goto 4 else goto 5.
4. Calculate the average opinion of the *q*-panel influencing the *i*-th spinson:
  $$\begin{array}{c} M_{i}\left(t\right)=\frac{1}{q}\sum_{j=1}^{q}S_{j}\left(t\right); \end{array}$$ (5)
  goto 6.
5. Calculate the average opinion of the *q*-panel influencing the *i*-th spinson:
  $$\begin{array}{c} M_{i}\left(t\right)=\frac{1}{q}\sum_{j=1}^{q}\sigma_{i}\sigma_{j}S_{j}\left(t\right) \end{array}$$ (6)
  and goto 6.
6. If *M*_*i*_(*t*) = 1 then *S*_*i*_(*t* + 1) = 1, else if *M*_*i*_(*t*) = −1 then *S*_*i*_(*t* + 1) = −1, else nothing changes and goto 1.

Unless stated otherwise “a random choice” refers to a draw from a uniform distribution.

As usual, a single Monte Carlo step consists of 2*N* updates, i.e. *τ* = 2*Nt*, which means that one time unit corresponds to the mean update time of a single individual. As can be seen, we have a model in which spinsons from clique 1 follow others and therefore we call them “followers”. On the other hand, spinsons from clique 2 follow only spinsons from their own group and anticonform to agents from the other group and therefore we call them “snobs”. Within the original, symmetrical formulation in [22], the polarization was observed above the critical value of cross links, L*(*q*). The question is: how will the results change under the modification we make in this paper?

### A model with annealed cross links

We will investigate the model with quenched links using only Monte Carlo simulations because for a quenched network we are not able to solve the model analytically. However, the theoretical description simplifies significantly if we replace the quenched network by an “annealed network”. In the latter, instead of having stable *LN*^2^ links between the cliques, we introduce a probability, *p*, that a cross link exists. Within such a modification we are able to write down equations that describe the time evolution of the public opinion. But before doing this let us describe the algorithm for a single Monte Carlo update in the model with annealed links:

1. Randomly choose one spinson, say the *i*-th spinson, from the 2*N* spinsons in the system; goto 2.
2. The *i*-th spinson chooses *q* neighbors from the whole population (*q*-panel): *S*_1_, …, *S*_*q*_. Each of the *q* spinsons is chosen independently, i.e. a repeated choice of the same node is allowed (note, that the asymptotic results would not change if the *q*-panel consisted of *q* different spinsons). Nodes from the *i*-th spinson’s clique are chosen with probability 1 − *p* and nodes from the other clique are chosen with probability *p*; goto 3.
3. If the *i*-th spinson belongs to clique 1 then goto 4 else goto 5.
4. Calculate the average opinion of the *q*-panel influencing the *i*-th spinson:
  $$\begin{array}{c} M_{i}\left(t\right)=\frac{1}{q}\sum_{j=1}^{q}S_{j}\left(t\right); \end{array}$$ (7)
  goto 6.
5. Calculate the average opinion of the *q*-panel influencing the *i*-th spinson:
  $$\begin{array}{c} M_{i}\left(t\right)=\frac{1}{q}\sum_{j=1}^{q}\sigma_{i}\sigma_{j}S_{j}\left(t\right) \end{array}$$ (8)
  and goto 6.
6. If *M*_*i*_(*t*) = 1 then *S*_*i*_(*t* + 1) = 1, else if *M*_*i*_(*t*) = −1 then *S*_*i*_(*t* + 1) = −1, else nothing changes and goto 1.

For such a reformulated (annealed) model we are able to derive the limiting dynamical system for *N* → ∞ in scaled time $t=\frac{\tau }{2N}$. We first calculate transition probabilities and then derive the asymptotic dynamical system expectations. Let $\Delta_{N}:=\frac{1}{2N}$ and $N_{i}^{+}\left(t\right)$ be the number of nodes in state +1 at time *t* in clique *i* = 1, 2. Using the corresponding densities, $c_{i}\left(t\right)=N_{i}^{+}\left(t\right)/N$, we can write down the transition probabilities:

- For clique 1:
  $$\begin{array}{ccc} \gamma_{+} & = & Pr\left\{N_{1}^{+}\left(t+\Delta_{N}\right)=N_{1}^{+}\left(t\right)+1\right\}=\frac{1}{2}\left(1-c_{1}\right){\left(\left(1-p\right)c_{1}+pc_{2}\right)}^{q},\\ \gamma_{-} & = & Pr\left\{N_{1}^{+}\left(t+\Delta_{N}\right)=N_{1}^{+}\left(t\right)-1\right\}=\frac{1}{2}c_{1}{\left(\left(1-p\right)\left(1-c_{1}\right)+p\left(1-c_{2}\right)\right)}^{q},\\ \gamma_{0} & = & Pr\left\{N_{1}^{+}\left(t+\Delta_{N}\right)=N_{1}^{+}\left(t\right)\right\}=1-\left(\gamma_{+}+\gamma_{-}\right), \end{array}$$ (9)
- and for clique 2:
  $$\begin{array}{ccc} \gamma_{+} & = & Pr\left\{N_{2}^{+}\left(t+\Delta_{N}\right)=N_{2}^{+}\left(t\right)+1\right\}=\frac{1}{2}\left(1-c_{2}\right){\left(\left(1-p\right)c_{2}+p\left(1-c_{1}\right)\right)}^{q},\\ \gamma_{-} & = & Pr\left\{N_{2}^{+}\left(t+\Delta_{N}\right)=N_{2}^{+}\left(t\right)-1\right\}=\frac{1}{2}c_{2}{\left(\left(1-p\right)\left(1-c_{2}\right)+pc_{1}\right)}^{q},\\ \gamma_{0} & = & Pr\left\{N_{1}^{+}\left(t+\Delta_{N}\right)=N_{1}^{+}\left(t\right)\right\}=1-\left(\gamma_{+}+\gamma_{-}\right). \end{array}$$ (10)

The factor $\frac{1}{2}$ in the above formulas arises from the fact that we have 2 cliques of equal size, hence the probability for choosing a node from one given clique is 1/2. We could also consider unequal clique sizes, but this would require an additional parameter and make the analysis more technical. Using probabilities Eqs (9) and (10) we can simulate trajectories *c*_1_(*t*) and *c*_2_(*t*), which can be easily converted to trajectories of the public opinion using the following relation:

$$\begin{array}{c} m_{i}\left(t\right)=2c_{i}\left(t\right)-1. \end{array}$$ (11)

Until now we have been dealing with random variables *c*_*i*_(*t*), but we can also write the evolution equations of the corresponding expected values. For *N* → ∞ we can safely assume that random variables *c*_*i*_(*t*) localize to the expectation values:

$$\begin{array}{ccc} \frac{c_{1}\left(t+\Delta_{N}\right)-c_{1}\left(t\right)}{\Delta_{N}} & = & \left(1-c_{1}\right){\left(\bar{p}c_{1}+pc_{2}\right)}^{q}-c_{1}{\left(\bar{p}\left(1-c_{1}\right)+p\left(1-c_{2}\right)\right)}^{q},\\ \frac{c_{2}\left(t+\Delta_{N}\right)-c_{2}\left(t\right)}{\Delta_{N}} & = & \left(1-c_{2}\right){\left(\bar{p}c_{2}+p\left(1-c_{1}\right)\right)}^{q}-c_{2}{\left(\bar{p}\left(1-c_{2}\right)+pc_{1}\right)}^{q}, \end{array}$$ (12)

where $\bar{p}=1-p$. Taking the limit *N* → ∞, we get the following continuous time dynamical system:

$$\begin{array}{ccc} \frac{dc_{1}}{dt} & = & \left(1-c_{1}\right){\left(\bar{p}c_{1}+pc_{2}\right)}^{q}-c_{1}{\left(\bar{p}\left(1-c_{1}\right)+p\left(1-c_{2}\right)\right)}^{q},\\ \frac{dc_{2}}{dt} & = & \left(1-c_{2}\right){\left(\bar{p}c_{2}+p\left(1-c_{1}\right)\right)}^{q}-c_{2}{\left(\bar{p}\left(1-c_{2}\right)+pc_{1}\right)}^{q}. \end{array}$$ (13)

The set of Eq (13) can be solved numerically. From the condition

$$\begin{array}{c} \frac{dc_{1}}{dt}=\frac{dc_{2}}{dt}=0, \end{array}$$ (14)

we can obtain the stationary value of *c*_1_ and *c*_2_ and from Eq (13) the time evolution of these variables.

## Results

We will present results for two models, each characterized by three parameters:

- a model with quenched cross links, where *L* ∈ [0, 1] is the fraction of cross links, *q* is the number of agents in the group of influence (which varies from 2 to 7 in agreement with the empirical results [43, 44]) and *N* is the size of the clique (here we present results only for *N* = 10^2^, 10^3^, 10^4^),
- a model with annealed cross links, where *p* ∈ [0, 1] is the probability of a cross link and *q* and *N* are the same as above.

The model with quenched cross links will be investigated by Monte Carlo (MC) simulations and the model with annealed cross links by MC simulations using transition probabilities given by Eqs (9) and (10), as well as by numerical solutions of Eq (13).

It is worth noting, that parameter *p* can be easily related to *L*. In fact, from now on we will use parameter *p* for both models. Because *p* denotes the probability that a single individual will choose a neighbor from the other clique we can write:

$$\begin{array}{c} p=\frac{LN}{LN+N}=\frac{L}{L+1}, \end{array}$$ (15)

where *LN* is the expected number of the neighbors from the other clique, and *LN* + *N* is a total number of neighbors for a single individual. Using relation Eq (15), results for both models can be easily compared if only *p* ≤ 0.5. For *p* > 0.5 the cross links are more probable than links inside the groups and *L* > 1, which is impossible if we assume that groups are represented by cliques (complete graphs) and we deal with unweighted links. However, it would be possible to introduce bias and assume that cross links have different weights than links inside a clique and then it would be possible to obtain *p* > 0.5. Again, this modification requires at least one additional parameter and makes the analysis more technical. Therefore, similarly as in the case of unequal cliques, we leave it for a future study.

**Fig 3:**
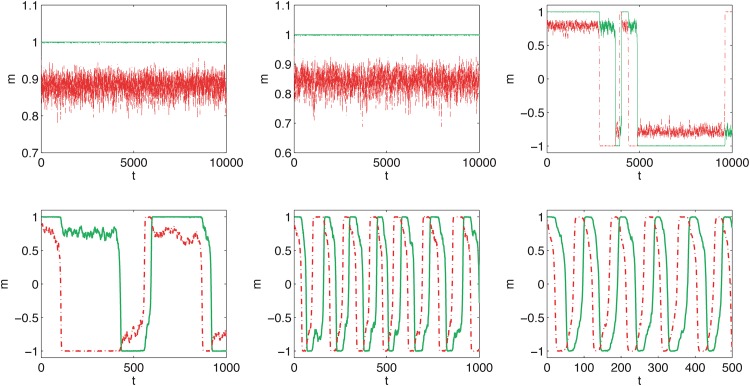
Sample evolution of the opinion for quenched cross links. Results for the snobs are presented by the red dashed lines and for the followers by the solid green lines. The probability of cross links, *p*, in each panel is different and increases from the top left panel to the right bottom: p = 0.15, 0.16, 0.16667, 0.17, 0.18, 0.19 (which corresponds to L = 0.176, 0.190, 0.200, 0.205, 0.220, 0.235). The size of each clique is *N* = 1000 for all panels.

**Fig 4:**
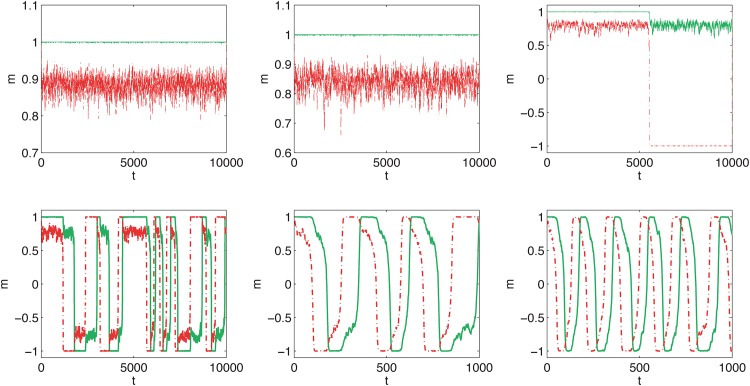
Sample evolution of opinion for annealed cross links. Results for the snobs are presented by the red dashed lines and for the followers by the solid green lines. The probability of cross links, *p*, in each panel is different and increases from the top left panel to the right bottom: p = 0.15, 0.16, 0.16667, 0.17, 0.18, 0.19 (the same values as in Fig 3). The size of each clique is *N* = 1000 for all panels, as in Fig 3.

We start with presenting trajectories, which represent how popularity—measured e.g. in terms of public opinion or market share of a given product—change over time. We investigate the system with two cliques of size *N* = 1000 and consider ordered initial conditions, i.e. *m*_1_(0) = 1 and *m*_2_(0) = 1, which corresponds to the situation in which everybody likes/uses the same type of product, e.g. all women wear short skirts. For the fixed size of the system both models are described only by two parameters *q* and *p*. As can be seen in Figs 3 and 4, the time evolution of the public opinion is similar in both models:

- For a small probability of cross links, *p* < p*, both cliques are almost fully ordered in the same direction: *m*_1_(*t*) > 0 and *m*_2_(*t*) > 0. Moreover, opinion *m*_2_(*t*) < *m*_1_(*t*) fluctuates strongly around the average <*m*_2_(*t*)> and *m*_1_(*t*) is almost constant.
- Above the critical probability of cross links, i.e. for *p* > p*, recurring cycles are observed. The period of the cycle decreases with *p*. Moreover, near the critical point the cycles are irregular and hence a change of fashion is difficult to predict.

**Fig 5:**
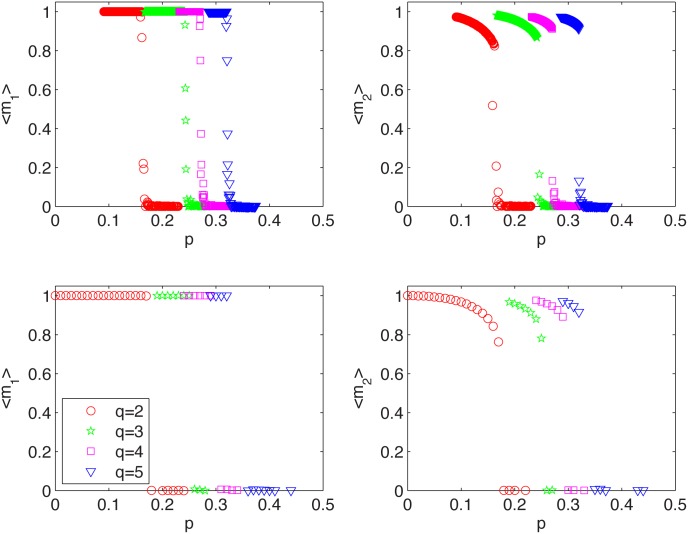
Dependence between the average stationary opinion and the fraction of cross links. Average opinion as a function of the cross links in the stationary state for the followers (left panels) and the snobs (right panels) for the model with quenched cross links (upper panels) and the model with annealed cross links (bottom panels). These results do not depend on the system size and have been found to be the same for *N* = 10^2^, 10^3^, 10^4^.

There is a good theoretical reason, which needs more technical details and will not be discussed here, to believe that for *N* → ∞ the annealed variant of the model should converge to the quenched variant. Indeed, we can see from Figs 3 and 4 that threshold p* is roughly the same for both models. This can be seen clearly if we plot the time average of public opinion <*m*> as a function of *p*. For *p* > p* it should be zero, because the opinion is as often negative as positive. Indeed, we can see in Fig 5 that there is critical value of *p* = p*(*q*) above which recurring cycles appear. It is also seen that p*(*q*) increases with *q*, which has been also observed for the one-clique version of the *q*-voter with anticonformity [24].

To see the difference between the models we have to observe the shapes of the trajectories—immediately we can see that the period of the cycle for the model with quenched cross links (Fig 3) is significantly smaller than for the model with annealed cross links (Fig 4). To measure this difference quantitatively we can calculate the average time of the cycle. Near the critical point (see the top left and bottom right panels in Figs 3 and 4), the cycles are strongly irregular, which means that the period varies. If we measure the period, i.e. *T* such that *m*(*t*) = *m*(*t* + *T*), for the first cycle it will be significantly different than the period for the next cycle. However, it is seen that for larger values of *p* the period is almost constant and therefore the average, <*T*>, is a good measure of the rate of change.

**Fig 6:**
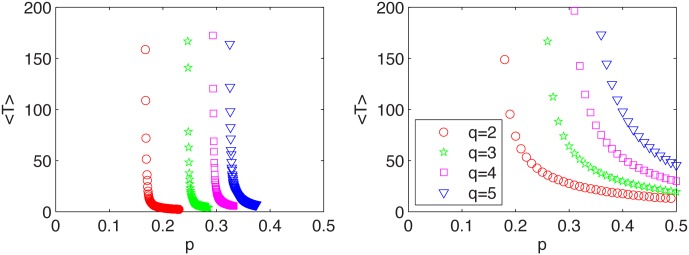
The average time of a cycle. Results for the model with quenched (left panel) and with annealed cross links (right panel).

In Fig 6 it can be seen that period *T* decreases with *p* and is shorter for the model with quenched cross links, if only *p* is distinctly larger than p*. These features have been already seen qualitatively in Figs 3 and 4. This is an interesting result from the social point of view, because it seems that presently social networks are more annealed than in the past. Nowadays we use the Internet and social media, which may result in an increasing randomness of cross links. According to our model, this should result in slower recurring fashion cycles (larger *T*). On the other hand, we may now expect faster recurring fashion cycles (smaller *T*), because the probability of interactions between communities, *p*, is now larger than in the past. Thus, there is a competition of two factors—more changes and randomness in the social network (annealing factor) increases the period of a fashion cycle, but a larger number of connections between communities decreases *T*. The question is which of the two factors wins.

There are several more or less reliable hypotheses, including famous Laver’s law of fashion [50] or pop culture’s 40-year cycle of nostalgia, that suggest a constant value of *T*. If indeed *T* is constant then all forces/factors influencing the recurring fashion cycle must act contrary to each other and balance out. However, all these observations are merely speculations. We do not have a firm empirical evidence that presently social networks are more annealed and *p* increases over time. Moreover, we have not found any reliable long-time empirical data that would clearly answer whether the period of the fashion cycle decreases, increases or remains constant. Therefore collecting and analyzing relevant empirical data is the challenge for the future.

We have to note, that period *T* is shorter for the quenched model only for *p* distinctly larger than p* (compare Figs 3 and 4; see also Fig 6). However, for *p* close to p* the period is shorter for the annealed model. Therefore, if the social system is near the critical state we should expect more frequent changes for the annealed network. Moreover, for *p* → p* changes in fashion are strongly irregular and therefore hard to predict. In such a case it is even hard to speak about any period of a cycle.

## Additional comments on the annealed model

As we have already mentioned, it makes no sense to consider *p* > 0.5 if we use a model without any bias, i.e. when all links (cross links and intra-links) have the same weight. However, if we are interested in the annealed model itself, we can consider any value of *p* ∈ [0, 1], which can be instructive, particularly if we take into account that present social networks are not as stable is in the past.

**Fig 7:**
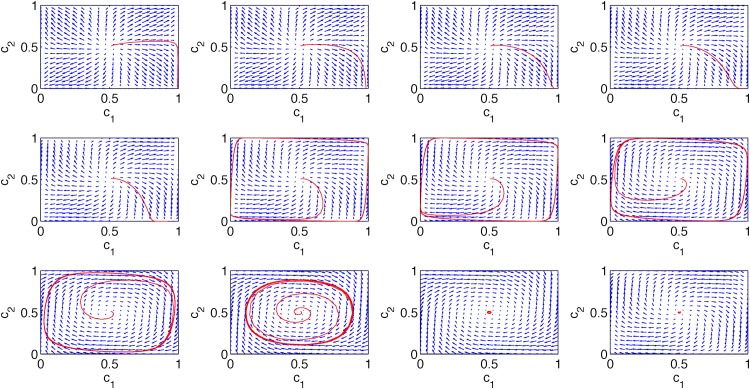
The phase portraits for the model with annealed cross links and *q* = 2. Blue arrows indicate the direction of flows in the phase plane and the red line is a sample average trajectory from disordered initial conditions *c*_1_(0) = *c*_2_(0) = 0.5001. The probability of a cross link, *p*, in each panel is different and increases from the top left to the bottom right panel: p = 0.08, 0.10, 0.12, 0.14, 0.16, 0.18, 0.20, 0.24, 0.30, 0.40, 0.50, 0.60. The size of each clique is *N* = 10^4^ for all panels.

The annealed model has a big advantage over the quenched model—it is much simpler for analytical treatment. Here we present results of numerical solutions of Eq (13) that were obtained using the fourth order Runge-Kutta scheme. Until now we have discussed only one type of the initial conditions but using the flow diagrams in the phase space we are able to see the behavior for arbitrary initial conditions (see Fig 7).

**Fig 8:**
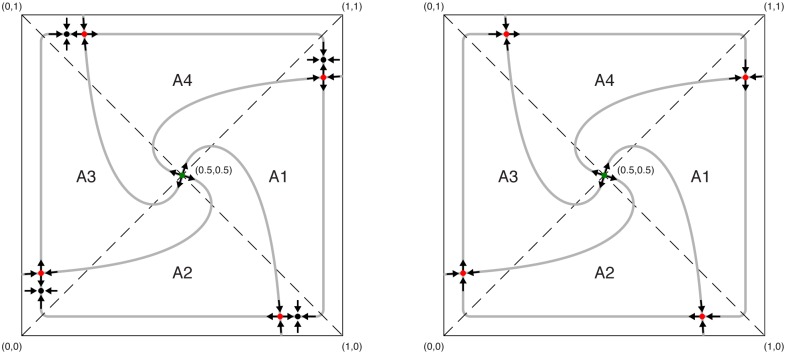
Schematic representation of the system behavior near the critical point in the phase plane. The situation for *p* < p* is shown in the left panel. There are 8 fixed points: 4 stable (black) and 4 hyperbolic saddle points (red). Moreover, we have an additional unstable point at (0.5, 0.5); A1-A4 are the basins of attraction. In the right panel we see the system at the critical point *p* = p*. The saddle and stable fixed points merge, hence we have 4 fixed points and a so-called heteroclinic cycle. For *p* > p* the fixed points disappear and only the stable limit cycle is left.

From Fig 7 we can see that indeed we have a critical value of *p* above which the limit cycle appears and the period decreases with *p*. However, additionally we see that below the critical point the system can reach different fixed points depending on the initial conditions. We conjecture on topological grounds and the structure of the vector field plots (Fig 7) that the saddle node heteroclinic bifurcation appears at p* [51]. It is not easy to see this in Fig 7, therefore we show schematically the behavior of the system around *p* = p* in Fig 8.

For *p* < p* (see the left panel in Fig 8) there are 8 fixed points (4 stable ones and 4 hyperbolic saddle points). At the bifurcation point *p* = p* (see the right panel in Fig 8) the unstable and stable fixed points merge, hence one has 4 fixed points and a so-called heteroclinic cycle. For *p* > p* the fixed points disappear and only the stable limit cycle is left. These type of bifurcations appear generically in one parameter families of flows.

**Fig 9:**
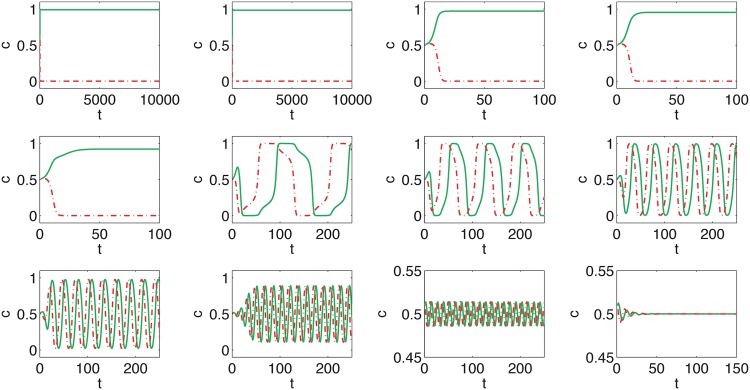
Average trajectories for the model with annealed cross links and *q* = 2. Results for snobs are presented by the red dashed lines and for the followers by the solid green lines. The probability of cross links, *p*, is different in each panel and increases from the top left to the bottom right panel: p = 0.08, 0.10, 0.12, 0.14, 0.16, 0.18, 0.20, 0.24, 0.30, 0.40, 0.50, 0.60. The size of each clique *N* = 10^4^ for all panels.

Because the configuration space may be more intuitive for many readers, we additionally present the results from Fig 7 in the (*c*_*i*_, *t*) plane; see Fig 9. In this representation we lose the information about the role of initial conditions. Instead interesting phenomena are clearly seen for large values of *p*—fading cycles appear and the system eventually reaches a disordered fixed point, *c*_1_ = *c*_2_ = 0.5, in which both products/opinions are equally popular in the system. In fact, the same phenomenon could be seen also in Fig 7, but a more zoomed-in picture would be needed (pay attention to the scale in Figs 7 and 9).

This behavior means that there is another critical point below which the system reaches a limit cycle and above which a stable fixed point $\left(c_{1},c_{2}\right)=\left(\frac{1}{2},\frac{1}{2}\right)$. The appearance of an attractive limit cycle of a smooth dynamical system is actually the result of a Hopf bifurcation at the fixed point $\left(c_{1},c_{2}\right)=\left(\frac{1}{2},\frac{1}{2}\right)$, which takes place for the critical value $p_{bif}=\frac{q-1}{q}.$ In the special case of a 2 dimensional system a Hopf bifurcation occurs when a fixed point—in our case the one at $\left(\frac{1}{2},\frac{1}{2}\right)$ − looses stability at a critical parameter value in such a way that the linearized system at the fixed point has two complex conjugate eigenvalues. These eigenvalues are purely imaginary at the bifurcation value and have positive/negative real part above/below the bifurcation value. Furthermore, some non-degeneracy conditions have to be met; for details see [52]. To check if the conditions of Hopf’s theorem apply we first compute the Jacobian matrix at the fixed point $\left(c_{1},c_{2}\right)=\left(\frac{1}{2},\frac{1}{2}\right).$

Recall that the dynamics is given by Eq (13). Some straightforward computation gives for Jacobian *J* at $\left(\frac{1}{2},\frac{1}{2}\right)$ the following matrix:

$$\begin{array}{c} J=\left(\begin{array}{cc} {\left(\frac{1}{2}\right)}^{q-1}\left(q\left(1-p\right)-1\right) & qp{\left(\frac{1}{2}\right)}^{q-1}\\ -qp{\left(\frac{1}{2}\right)}^{q-1} & {\left(\frac{1}{2}\right)}^{q-1}\left(q\left(1-p\right)-1\right) \end{array}\right), \end{array}$$ (16)

which is of the form

$$\begin{array}{c} \left(\begin{array}{cc} a & b\\ -b & a \end{array}\right) \end{array}$$ (17)

and the eigenvalues are *a* − *ib*, *a* + *ib*. For a Hopf bifurcation to happen we need two conjugate purely imaginary eigenvalues, hence *a* = 0. This is equivalent to *q* − 1 − *qp* = 0 and

$$\begin{array}{c} p=p_{bif}\left(q\right)=\frac{q-1}{q}. \end{array}$$ (18)

It is easy to check that all conditions of Hopf’s theorem are met and hence the loss of stability of the central fixed point creates an attractive cycle.

**Fig 10:**
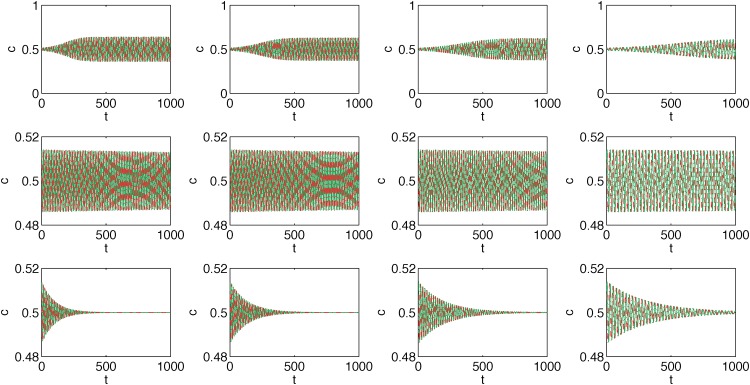
Average trajectories for the model with annealed cross links and several values of *q*. Each column corresponds to a different value of *q*, from left to right *q* = 2, 3, 4, 5. Results for the snobs are presented by the red dashed lines and for the followers by the solid green lines. Upper panels correspond to the probability of cross links *p* = *p*_*bif*_(*q*) − *ϵ*, middle panels to *p* = *p*_*bif*_(*q*) and bottom panels to *p* = *p*_*bif*_(*q*) + *ϵ*, where *ϵ* = 0.01. Note the difference in ranges on the vertical axis that corresponds to *c*—in the upper panels the range is much larger, i.e. [0, 1] than in the middle and bottom panels. Values of *p*_*bif*_, agree with the derived analytical Formula (18), i.e. $p_{bif}\left(2\right)=\frac{1}{2}$ (first column), $p_{bif}\left(3\right)=\frac{2}{3}$ (second column), $p_{bif}\left(4\right)=\frac{3}{4}$ (third column), $p_{bif}\left(5\right)=\frac{4}{5}$ (fourth column). The size of each clique is *N* = 10^4^ for all panels.

The above results mean that for *p* < *p*_*bif*_(*q*) a fixed point $\left(c_{1},c_{2}\right)=\left(\frac{1}{2},\frac{1}{2}\right)$ is repelling and the system reaches a limit cycle, whereas for *p* > *p*_*bif*_(*q*) a fixed point $\left(c_{1},c_{2}\right)=\left(\frac{1}{2},\frac{1}{2}\right)$ is attracting and the system eventually reaches this point. This is clearly seen in Fig 10. For *p* = *p*_*bif*_ − *ϵ* the system reaches a limit cycle and for *p* = *p*_*bif*_ − *ϵ* the system reaches a fixed point $\left(\frac{1}{2},\frac{1}{2}\right)$. Moreover, values of *p*_*bif*_ agree with the derived analytical Formula (18), i.e. $p_{bif}\left(2\right)=\frac{1}{2}$, $p_{bif}\left(3\right)=\frac{2}{3}$, $p_{bif}\left(4\right)=\frac{3}{4}$, $p_{bif}\left(5\right)=\frac{4}{5}$.

## Conclusions

With some exceptions [12, 13], in most models of fashion and fads two types of social response are taken into account—conformity and anticonformity, sometimes referred to as imitation and distinction [9] or coordination and anti-coordination [53]. Some models take into account also the influence of certain exogenous factors, like popularity of the product [54]. We are aware that exogenous factors including popularity or advertisement are important in creating fashion, but it seems that they are not necessary, what has been shown in many other models [12, 13, 53]. Our aim was not to propose the final and complete model of the recurring fashion cycles but rather to show that a small modification of the model without cycles [22] can lead to the appearance of a periodic behavior. Moreover, we wanted to examine what is the role of stable connections between cliques. Therefore we have considered two versions of the same model—quenched (parameterized by fraction *L* of fixed inter-links) and annealed (parameterized by probability *p* of inter-links). For *p* ≤ 0.5 we have derived the relation between parameters *L* and *p* that allows to compare both models and show that the critical value of inter-connections is the same for the quenched and annealed schemes. However, the period of the cycles has been found to be shorter for the quenched model, i.e. for static social networks, if *p* is distinctly larger than p*. On the other hand, if *p* is close to p* then the period is shorter for the annealed model. Moreover, near the critical point the cycles are irregular and a change of fashion is difficult to predict.

In case of the annealed model we were able to write down equations describing the evolution of the system and provide deeper theoretical analysis. We conjectured on topological grounds that the so-called saddle node heteroclinic bifurcation appears at p*. Moreover, we have shown analytically that for *p* ≤ 0.5 there is another critical point below which the system reaches a limit cycle and above which a stable fixed point $\left(c_{1},c_{2}\right)=\left(\frac{1}{2},\frac{1}{2}\right)$. The appearance of an attractive limit cycle of the smooth dynamical system is actually the result of a Hopf bifurcation at the fixed point $\left(c_{1},c_{2}\right)=\left(\frac{1}{2},\frac{1}{2}\right)$, which takes place for the critical value $p_{bif}=\frac{q-1}{q}$. This result means that if the influence from the other clique is large enough, i.e. *p* > *p*_*bif*_, there is a possibility of a stale-mate state, in which there is no fashion.

We are aware that bringing the model closer to reality would require further development. For example, one could consider cliques of different sizes. Another modification, also mentioned in the paper, could be to assign weights to links (for example *ω* for cross links and 1 − *ω* for intra-links). Within such a modification, it makes sense to compare both models also for *p* > 0.5. However, both modifications require additional parameters and would make the analysis more technical. Moreover, they do not provide many more insights. Therefore, for the sake of clarity of presentation, they were left for a more specialized journal.
